# Novel point mutations in the *ERG11* gene in clinical isolates of azole resistant *Candida* species

**DOI:** 10.1590/0074-02760150400

**Published:** 2016-03

**Authors:** Danielly Beraldo dos Santos Silva, Luana Mireli Carbonera Rodrigues, Adriana Araújo de Almeida, Kelly Mari Pires de Oliveira, Alexéia Barufatti Grisolia/

**Affiliations:** 1 Universidade Federal da Grande Dourados, Dourados, MS, Brasil; 2 Universidade Federal de Mato Grosso do Sul, Campo Grande, MS, Brasil

**Keywords:** yeasts, Candida krusei, voriconazole, 14α-demethylase, Y166S

## Abstract

The azoles are the class of medications most commonly used to fight infections caused by *Candida* sp. Typically, resistance can be attributed to mutations in *ERG11* gene (CYP51) which encodes the cytochrome P450 14α-demethylase, the primary target for the activity of azoles. The objective of this study was to identify mutations in the coding region of the*ERG11* gene in clinical isolates of *Candida*species known to be resistant to azoles. We identified three new synonymous mutations in the *ERG11* gene in the isolates of *Candida glabrata* (C108G, C423T and A1581G) and two new nonsynonymous mutations in the isolates of *Candida krusei* - A497C (Y166S) and G1570A (G524R). The functional consequence of these nonsynonymous mutations was predicted using evolutionary conservation scores. The G524R mutation did not have effect on 14α-demethylase functionality, while the Y166S mutation was found to affect the enzyme. This observation suggests a possible link between the mutation and dose-dependent sensitivity to voriconazole in the clinical isolate of *C. krusei*. Although the presence of the Y166S in phenotype of reduced azole sensitivity observed in isolate *C. krusei*demands investigation, it might contribute to the search of new therapeutic agents against resistant *Candida* isolates.

In Latin American countries, particularly Brazil, *Candida tropicalis* is responsible for 20-24% of all haematogenous infections (Nucci & Colombo 2007, Pfaller & Diekema 2007). It is most commonly seen in patients with neutropenia, diabetes mellitus, and in elderly patients (Sipsas et al. 2009). *Candida glabrata* and *Candida krusei* are the predominant nosocomial fungal pathogens in patients with haematologic malignancies or those undergoing bone marrow transplantation (Goldman et al. 1993, Nucci & Colombo 2007, Pfaller & Diekema 2007).

In the previous decades, there have been many cases of resistance to antifungal agents used in the prophylaxis and treatment of infections caused by *Candida*species (Jiang et al. 2012, Almeida et al. 2013). Mutations and increased expression of genes encoding enzymes responsible for the biosynthesis of ergosterol (Vandeputte et al. 2005, Barker & Rogers 2006) have been identified as the molecular mechanisms responsible for the development of azole resistance in *Candida* species (Barker & Rogers 2006, Berila et al. 2009, Ge et al. 2010, Carvalho et al. 2013).

The azoles, a major class of antifungal compounds, interfere with the ergosterol biosynthesis pathway in fungal membranes by inhibiting the cytochrome P450-dependent enzyme 14α-demethylase (Erg11p or 14DM), synthesised by the *ERG11* gene. Thus, mutations resulting in the increased expression of the *ERG11* gene could confer the yeast species with resistance to azoles by decreasing their drug binding affinity (Barker & Rogers 2006).

Several mutations are clustered into three hot spot regions in *ERG11*gene ranging from amino acids (aa) 105-165, 266-287, and-488 from *Candida albicans*, those regions were associated which *Candida*species resistant to azoles (Marichal et al. 1999, Perea et al. 2001, Chau et al. 2004, Vandeputte et al. 2005, Morio et al. 2010, Flowers et al. 2015, Grossman et al. 2015, Tan et al. 2015).

Vandeputte et al. (2005) found a missense mutation (Y132F) in strains of *C. tropicalis* resistant to fluconazole, which had been previously reported in *C. albicans* by Chau et al. (2004), conferring resistance to this drug. Carvalho et al. (2013), when investigating mutations on the *ERG11* gene in clinical isolates of *C. albicans*, *C. glabrata*, and *C. tropicalis*previously evaluated by fluconazole-susceptibility tests, have identified 14 different missense mutations, five of which had not been previously described, being that one new L321F mutation was identified in *C. albicans* resistant to fluconazole.

Therefore, the search for mutations in the *ERG11* gene in clinically relevant *Candida* species can provide a better understanding of the molecular mechanisms involved in resistance to antifungal agents and aid in epidemiological research. In addition, the genetic and molecular characterisation of resistant *Candida* species could help in the search for new bioactive molecules with antifungal activity. Therefore, the objective was to identify mutations in the coding region of the *ERG11* gene in clinical isolates of*Candida* species known to be resistant to azoles.

## MATERIALS AND METHODS

*Selection and growth* - The 14 clinical isolates of *C. glabrata*, *C. krusei*, and *C. tropicalis* belonged to the Mycology Collection of the Applied Microbiology Laboratory from Federal University of Grande Dourados, Brazil. The antifungal sensitivity was determined by the broth microdilution method in accordance with the rules of Clinical and Laboratory Standards Institute documents M27-A3 and M27-S4 (CLSI 2008a, 2012). The antifungals tested were fluconazole, itraconazole, and voriconazole.

The isolates were grown on Sabouraud dextrose agar (Difco, USA) and on CHROMagar*Candida* (Difco) to ensure purity and viability. Susceptibility cut-off points for fluconazole, itraconazole, and voriconazole were established according to the supplement M27-S3 and M27-S4 (CLSI 2008b, 2012). American Type Culture Collection (ATCC) strains of *C. glabrata* (ATCC 90030),*C. krusei* (ATCC 6258), and *C. tropicalis* (ATCC 750) were used as reference in the analysis.

*DNA extraction and polymerase chain reaction (PCR)* - The DNA of isolates and reference strains was extracted from three colony-forming units (2.40 × 10^7^ cell/cm^3^) reactivated and grown in Sabouraud dextrose broth using the YeaStar™ Genomic DNA Kit (Zymo Research Co, USA). The purity (260 nm/280 nm) and concentration (ng/µL) of the extracted DNA were determined using a nanophotometer (NanoPhotometer™ P-300 UV-Vis; Implen GmbH, Germany). The primers used for amplification of the coding region of the *ERG11* gene were described in Table I.

**TABLE I:** Primers used for amplification reaction of the *ERG11*gene coding region of *Candida* genus species

Species	Primers		Fragment (bp)	Reference
*C. tropicalis*	Ct-ERG11-1F	TCTGACATGGTGTGTGTGTG	678	Vandeputte et al. (2005)
	Ct-ERG11-1R	ATTGATGCCATCAATGGCAG		
GenBank M23673.1	Ct-ERG11-2F	ATCCCACAGGCTTATTTGAAA	614	
	Ct-ERG11-2R	GGTCTCTTTCCTTGGTTTTG		
	Ct-ERG11-3F	TGCTGAAGAAGCTTATACCC	499	
	Ct-ERG11-3R	CAAGGAATCAATCAAATCTCTC		
	Ct-ERG11-3.1F	TGACGCTGCTCAAAGAAAGA	493	^*a*^
	Ct-ERG11-3.1R	ATGAGCATAACCGGCAGAAA		
	Ct-ERG11-4F	GGTGGTCAACATACTTCTGC	630	Vandeputte et al. (2005)
	Ct-ERG11-4R	AGCAGGTTCTAATGGTAAGG		
	Ct-ERG11-5F	AAACGGTGATAAGGTTCCAG	626	
	Ct-ERG11-5R	TCCCAAGACATCAAACCCTG		
*C. glabrata*	Cg-ERG11-0F	TCGGTCCATCTCTGTTTCTT	699	^*a*^
	Cg-ERG11-0R	GAACACTGGGGTGGTCAAGT		
GenBank EU219981.1	Cg-ERG11-1F	ACTACAATAACATGTCCACTGA	408	Carvalho et al. (2013)
	Cg-ERG11-1R	GGTGGTCAAGTGGGAGTAA		
	Cg-ERG11-2F	AGCTGCTTACTCCCACTTGACC	412	
	Cg-ERG11-2R	AGCTTGTTGGGCATGGTCTCTC		
	Cg-ERG11-3F	GCCCAACAAGCTATCTCTGGTA	418	
	Cg-ERG11-3R	TGTTTGGAATAGCGACATCTCTC		
	Cg-ERG11-4F	CCAAACACTTCCTACGTTGTCCC	424	
	Cg-ERG11-4R	GCATCTAGTACTTTTGTTCTGGATG		
*C. krusei*	Ck-ERG11-1F	CCTCTCTAGCAACAACAATGTCC	428	
	Ck-ERG11-1R	GCCCTTACCGAAAACAGGAGTG		
GenBank EU309502.1	Ck-ERG11-2F	ACTCCTGTTTTCGGTAAGGGCG	421	
	Ck-ERG11-2R	CACCGGCACGCTTTGTATTG		
	Ck-ERG11-3F	CGTGCCGGTGGTGAAATCAA	397	
	Ck-ERG11-3R	GGCCCTTTGGAACAATGTACGA		
	Ck-ERG11-4F	GTACATTGTTCCAAAGGGCCATT	410	
	Ck-ERG11-4R	GCTAGTTCTTTTGTCTTCTCTCC		

*a*: primer pairs proposed in this work obtained by the Software Primer 3.

The amplification reactions were performed using the MyCyclerTM Thermal Cycler (Bio-Rad, USA). The total reaction volume of 25 µL contained 12.5 µL of PCR Master Mix (Kapa Biosystems, South Africa), 1 µL of each primer (10 pmoles), and 2 µL of genomic DNA (10-20 ng). The PCR products were resolved using 2% agarose gel electrophoresis to assess their quality and integrity. The amplification program for all reactions was as follows: initial denaturation at 94ºC for 5 min, 30 denaturation cycles at 94ºC for 30 s, annealing at 50ºC for 40 s, extension at 72ºC for 50 s, followed by final extension at 72ºC for 10 min.

*Sequencing and data analysis* - The products of the PCR amplification were purified using isoamyl alcohol and sequenced in duplicate by the Sanger method (Sanger et al. 1977) on an ABI 3500 automated DNA sequencer (Applied Biosystems, USA) using the same primers used for PCR and BigDye Terminator cycle sequencing kit (Applied Biosystems). The sequences were read using the Sequencing Analysis v.5.3 software (Applied Biosystems).

For each isolate, a consensus sequence was established using the CAP3 software (Huang & Madan 1999). The consensus sequences were translated into their corresponding aa sequences using the ExPASy Translate Tool (web.expasy.org/translate/), taking into consideration the difference in translation of the CUN codons in yeasts (Moura et al. 2010). Alignment was performed using CLUSTALW 2.0 (Larkin et al. 2007) employing the sequences from this search and those available from GenBank with gene accessions L40389.1 and AY942647.1 (*C. glabrata*), KC542323.1 and KC542326.1 (*C. tro- picalis*), and DQ903901.1, DQ903902.1, DQ903903.1, DQ903904.1 and DQ903905.1 (*C. krusei*).

The aa phylogenetic tree was constructed using MEGA 6.0 (Tamura et al. 2013) by the neighbour-joining method (Saitou & Nei 1987), which follows the Poisson distribution model (Zuckerkandl & Pauling 1965). In addition, haplotype networks were generated using Network 4.1.1.2 program by the median-joining method (Bandelt et al. 1999) to analyse the relationship between haplotypes.

Point mutations in the aa sequence were located by aligning the sequence using CLUSTALW 2.0. Furthermore, the likelihood of functional impact of the nonsynonymous mutations found in this study on the 14α-demethylase enzyme activity was estimated. The substitution position-specific conservation evolutionary (subPSEC) score was calculated using the Protein ANalysis THrough Evolutionary Relationships tool (Thomas et al. 2003).

In accordance with Brunham et al. (2005), Thomas et al. (2003), and Thomas and Kejariwal et al. (2004), the subPSEC score estimates the likelihood of single aa substitution having a functional effect on the protein with based on the Hidden Markov model (HMM). SubPSEC scores are continuous values from 0 (neutral) to -10 (most likely to be deleterious). A cut-off score of -3, corresponding to a 50% probability that a score is deleterious (P_deleterious_ = 0.5), has been previously identified to be the cut-off point for functional significance.

*Sequences accessions* - The sequences obtained from different species of *Candida* were submitted to GenBank with the following accessions: KR998002, KR998003, KR998004, KR998005, KR998006, KR998007, KR998008, KR998009, and KR998010 from *C. glabrata*, KR998011, KR998012, KR998013, and KR998014 from *C. krusei*, and KR998015, KR998016, KR998017, and KR998018 from *C. tropicalis*.

## RESULTS

*Phylogenetic analysis* - The 14α-demethylase coding region of the*ERG11* gene was 1603 bp long for *C. glabrata*and 1587 bp long for *C. krusei* and *C. tropicalis.*Post translation, the length of the aa sequences was 533 for *C. glabrata* and 528 for *C. krusei* and *C. tropicalis.*

**Fig. 1:**
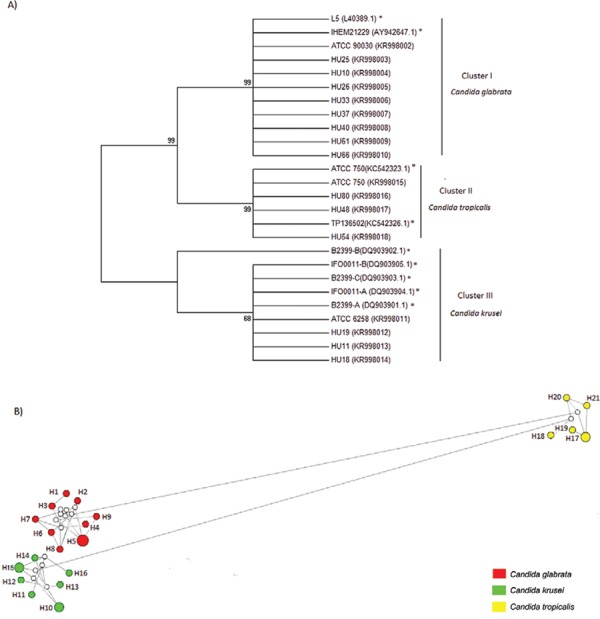
: phylogeny of the *ERG11* gene coding region of*Candida* species. A: the tree shown above was built from amino acids sequences of *ERG11* gene of strains of this study and sequences from GenBank, using the neighbour-joining method and evolutionary model of Poisson with 1,000 bootstrap replicates. Asterisks mean sequences obtained in the GenBank for comparison; B: network haplotype constructed by median-joining method based on *ERG11* gene sequence alignment of the isolates under study and GenBank sequences demonstrating the 21 haplotypes found for *Candida* species. The area of the haplotypes circles is proportional to its frequency. The length of the lines is related to the mutational steps separating each haplotype. The white dots are mean vectors representing hypothetical haplotypes introduced by the executed algorithm.

The average distance between the sequences was 0.3 (30%) (Fig. 1A). The phylogenetic tree showed bootstrap values of ≥ 70 on the main nodes. For clusters I and II, belonging to *C. glabrata*and *C. tropicalis*, intraspecific differences were not observed among the clinical isolates, ATCC reference strains, and sequences obtained from GenBank. For cluster III (belonging to *C. krusei*), a distance was observed among isolated IFO001 (accession DQ903902.1) and strains examined in this study.

Fig. 1B represents the relationship between the haplotypes found in the different species of *Candida* based on the mutations present on the *ERG11* gene sequence. The analysis revealed that the species studied belonged to different haplotypes due to difference in the coding region of the *ERG11* gene.

In the H5 haplotype were included the HU10, HU37, and HU61 strains (*C. glabrata*). These strains were isolated of uroculture and had four of total mutations found in *C. glabrata* (Table II). Two of the GenBank *C. krusei*sequences used in the comparisons were clustered in haplotype H10. We also observed that the strains that were dose dependent on the voriconazole HU11 and HU45 (*C. krusei*), isolated respectively of nasal swab and rectal swab, were clustered on the same haplotype (H15). In H17 haplotype were clustered the ATCC750 (accession KC542323.1) and the ATCC750 strains of *C. tropicalis* used in this study as reference. All other strains were clustered into haplotypes only ones.

**TABLE II:** Susceptibility profile and point mutations found in the*ERG11* gene coding region of clinical isolates of the *Candida* genus

Species	Isolate	GenBank	Isolation site	MIC (μg mL^-1^)			Point mutations

				Fluconazole	Itraconazole	Voriconazole	
*C. glabrata* (1,603 bp)	90030	KR998002	ATCC	(S)	(S)	(S)	A1581G^*a*^
	HU10	KR998003	Uroculture	8 (S)	≤ 0.125 (S)	1 (-)	C423T^*a*^, T768C, T1557A, A1581G^*a*^
	HU25	KR998004	Uroculture	8 (S)	≥ 1 (R)	1 (-)	C201G, T768C, A1023G, T1275C, T1557A, A1581G^*a*^
	HU26	KR998005	Uroculture	8 (S)	≥ 1 (R)	≥ 4 (-)	T768C, G927A, A1023G, T1557A, A1581G^*a*^
	HU33	KR998006	Rectal swab	8 (S)	≤ 0.125 (S)	≥ 4 (-)	T768C, A1023G, T1557A, A1581G^*a*^
	HU37	KR998007	Uroculture	8 (S)	≤ 0.125 (S)	≥ 4 (-)	C423T^*a*^, T768C, A1023G, T1275C, T1557A, A1581G^*a*^
	HU40	KR998008	Haemoculture	≥ 64 (R)	≥ 1 (R)	≥ 4 (-)	T768C, G927A, A1023G, T1275C, T1557A, A1581G^*a*^
	HU61	KR998009	Uroculture	16 (S)	≥ 1 (R)	1 (-)	C423T^*a*^, T768C, A1023G, T1275C, T1557A, A1581G^*a*^
	HU66	KR998010	Uroculture	16 (S)	≤ 0.125 (S)	≥ 4 (-)	C108G^*a*^, C423T^*a*^, T768C, A1023G, T1275C, T1557A, A1581G^*a*^
*C. krusei*(1,607 bp)	6258	KR998011	ATCC	(S)	(S)	(S)	T1389C, A1470C, G1570A^*b*^
	HU45	KR998012	Rectal swab	8 (-)	≤ 0.125 (S)	1 (DDS)	T642C, A756T, T1389C, A1470C, G1570A^*b*^
	HU11	KR998013	Nasal swab	≥ 64 (-)	≤ 0.125 (S)	1 (DDS)	T642C, A756T, T1389C, A1470C, G1570A^*b*^
	HU18	KR998014	Haemoculture	≥ 64 (-)	≤ 0.125 (S)	1 (DDS)	A497C^*b*^, T642C, T1389C, A1470C, G1570A^*b*^
*C. tropicalis* (1,587 bp)	750	KR998015	ATCC				N
	HU80	KR998016	Uroculture	8 (R)	≤ 0.125 (S)	≥ 4 (R)	T783C
	HU48	KR998017	Catheter tip	16 (R)	≤ 0.125 (S)	1 (R)	G1362A, T1554
	HU54	KR998018	Uroculture	8 (R)	≤ 0.125 (S)	1 (R)	G1362A

*a*: new synonymous mutation; *b*: new nonsynonymous mutations, A497C = Y166S and G1570A = G524R; DDS: dose dependent sensitivity; MIC: minimum inhibitory concentration; N: absent base substitution; R: resistant; S: sensitive; -: no significant evidence to determine the cut-off value for the species.

*Point mutations in the ERG11 gene* – Twenty-five different nucleotide changes were identified (17 transitions and 8 transversions) after inspecting all the evaluated sequences, including those obtained from GenBank. Twenty synonymous mutations (which do not alter the aa sequence of the protein) and two nonsynonymous mutations (which alter the aa sequence of the protein) were identified among the sequences obtained from the clinical isolates in this study. No insertions, deletions, or nonsense mutations were found.

The largest number of point mutations (11) was found in *C. glabrata*(Table II), where two of them (C678T and T1521A) were only found in isolate IHEM21229 (accession AY942647.1) used as a reference in this study. We found none mutations point in the strain L5 (accession L40389.1) used as a reference in this study. In case of *C. glabrata*, none of the mutations was found to alter the aa sequence of the 14α-demethylase. Three of the synonymous point mutations (C108G, C423T, and A1851G) found in this species have not been previously reported (Table II).

A single point mutation was identified in the HU54 and HU80 *C. tropicalis* isolates. Two mutations were identified in the HU48 isolated from *C. tropicalis* (Table II). Where three of them (T225C, G264A, and A395T) were only found in strain TP13650 (accession KC542326.1) used as a reference in this study. We found none mutations point in the strain ATCC 750 (accession KC542323.1). None of the nucleotide changes found in the sequences of *C. tropicalis* isolates altered the aa sequence of the resulting protein.

**Fig. 2:**
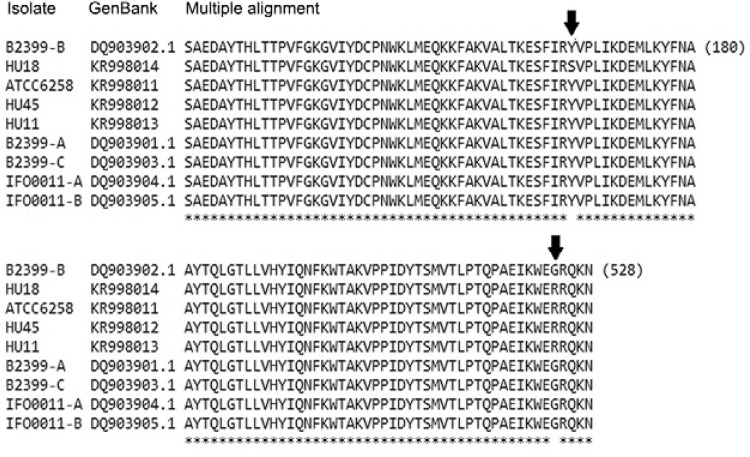
: multiple alignments of amino acid (aa) sequences of the*ERG11* gene of *Candida krusei*clinical isolates using the CLUSTALW 2.0 (Larkin et al. 2007). Aa conserved in all sequences are indicated by an asterisk. Arrows indicate novel nonsynonymous mutation (Y166S and G524R) found *C. krusei* strains.

Five synonymous mutations and two novel nonsynonymous mutations - Y166S and G524R (tyrosine for serine at position 166 and glycine for arginine at position 524 in the 14α-demethylase aa sequence) - were identified in the clinical isolates of*C. krusei* with dose dependent sensitivity to voriconazole (Fig. 2, Table II). We noted also, one nonsynonymous mutation, C44T, resulting in an aa change from alanine to valine in sequence of the strain IFO0011 (accession DQ903905.1).

The G524R mutation was observed in ATCC 6258 strain and in isolates with dose dependent sensitivity in this study, thus indicating that it may not be directly related to the susceptibility to azoles. However, the Y166S mutation was observed only in the isolate HU18, and not in the sequences obtained from GenBank, suggesting that it may induce a decrease in affinity to the azoles.

The likelihood that Y166S mutation may cause a deleterious effect on the function of the 14α-demethylase is estimated by P_deleterious_. The subPSEC score obtained was -4.08665, which corresponds to a P_deleterious_ of 0.74775, indicating that the mutation Y166S might be deleterious. The subPSEC score was not generated for the G524R mutation. This substitution occurred at a position that did not appear in the multiple sequence alignment. In most cases, these positions are not modelled by the HMMs simply because they do not appear in most of the related sequences; as a result, substitutions at these positions are not likely to be deleterious.

## DISCUSSION

The phylogenetic tree generated from the coding region sequences of the*ERG11* gene (Fig. 1A) indicates that this gene demonstrates high consistency and reliability in the analysis due to high bootstrap values making it favourable for inclusion in phylogenetic studies (Hillis & Bull 1993). The average distance between the sequences was found to be 30% in the compared residues. Although the ratio of differences is a good indicator of intraspecific variability, this value assumes that the probability of substitution is constant throughout the sequence. This might not always be the case, since there are regions more conserved than others (Russo et al. 2012).

Studying the coding region of *ERG11* made it possible to observe intraspecific variations in the *Candida* genus (division into taxonomic groups depicted in Fig. 1A), since change in the coding region is reflected in the form of a structural or functional change in protein (Li 2000). The same fact helped us observe differences between species using the haplotype network, suggesting that the gene under study could be used to identify intraspecific haplogroups. The haplotype analysis showed evidence that some clustered strains, besides sharing genetic similarities, also came from the same isolation site and have the same susceptibility profile, possibly indicating a clonal propagation.

The sequences of intraspecific aa obtained in this study are highly conserved. Owing to the degeneracy of the genetic code, rates of substitution were less. Hence, there was only a minor deviation in the topology of aa that determine protein function (Russo et al. 2012), as seen with 14α-demethylase (which maintains the integrity and function of the yeast plasma membrane) (Fig. 1A).

Mutations in the *ERG11* gene that change the aa sequence represent a major mechanism associated with resistance of *Candida* clinical isolates to azoles (Morio et al. 2010). Except for the C108G, C423T and A1581G mutations, synonymous mutations found in our isolates (Table II), have been previously described by Vandeputte et al. (2007) andBerila and Subik (2010). Berila and Subik (2010) observed that the T768C, A1023G, and T1557A mutations were present in all the studied isolates of *C. glabrata*, including the ones sensitive to azoles, implying that these changes might not play a role in conferring resistance to antifungal agents.

Forastiero et al. (2013) found the synonymous mutations T783C, T1554C, and G1362A in a clinical isolate (accession KC542326.1) of*C. tropicalis* resistant to fluconazole and voriconazole. Similarly, while investigating the mechanisms of resistance to azoles, Loeffler et al. (2000) evaluated 21 isolates and found five of them to have the T1554C mutation. Vandeputte et al. (2005) also found the T1554C mutation in the*ERG11* gene coding sequence in clinical isolates resistant to azoles.

These synonymous mutations in the *ERG11* gene in *C. tropicalis* have been identified several times in separate studies. Even though these mutations might not be directly responsible for the resistance to azoles, it is possible that the isolates with reduced susceptibility will be under selective pressure from the environment. With passage of time, the accumulation of these mutations in DNA sequence associated with other factors such as recombination could impact upon the enzyme functionality.

Lamping et al. (2009) had previously reported six of the synonymous mutations found in this study in clinical isolates of*C. krusei*. The nonsynonymous mutation G1470A (G524R), localised in C-terminal Erg11p, was found in the azole sensitive *C. krusei*reference strain ATCC 6258 and in isolates with dose dependent sensitivity to voriconazole.

Marichal et al. (1999) evaluated the effects of aa substitutions on subcellular sterol biosynthesis and azole sensitivity in*C. albicans*. They reported 16 synonymous and 12 nonsynonymous mutations; three of these mutations are associated with resistance and are located the C-terminal part of Erg11p. These high genetic polymorphisms suggest that lanosterol demethylase is highly permissive for structural changes. Several lines of evidence indicate that these aa changes do not contribute equally to azole resistance since the majority of these substitutions, instead of being randomly dispersed, are clustered into three hot spot regions ranging from aa 105-165, 266-287, and 405-488.

Fukuoka et al. (2003) constructed homology models of the CYP51s of *C. albicans* and *C. krusei*based on the crystal structure of CYP51 from *Mycobacterium tuberculosis.* The Erg11p in *C. albicans* has the same size in Erg11p *C. krusei*. Based on the results of Marichal et al. (1999) and Fukuoka et al. (2003), our results predicts that the replacement of aa at position 524 found in this study is located after the hot spot 3 (405 to 488), thus is not likely to be deleterious, and hence has no impact on the functionality of 14α-demethylase in *C. krusei*. However, the mutation A497C (Y166S) found in the HU18 isolate (*C. krusei*) with dose dependent sensitivity to voriconazole is located one position upstream within a hot spot ranging aa 105-165 from *C. albicans* and *C. krusei*, region within demonstrated associated which *Candida* species resistant to azoles (Marichal et al. 1999, Perea et al. 2001, Chau et al. 2004, Morio et al. 2010, Flowers et al. 2015, Grossman et al. 2015, Tan et al. 2015). The Y166S is neighbouring similar to that found E165Y by Marichal et al. (1999) in the *C. albicans* mutant, suggest that this mutation interferes with both itraconazole and fluconazole binding. Furthermore, the SubPSEC analysis results showed that Y166S might affect 14α-demethylase functionality.

The phenotype with reduced azole sensitivity observed in isolate HU18 (*C. krusei*) cannot be explained only by the presence of the A497C (Y166S) mutation. Our data show that point mutations leading to aa changes are a frequent event in *ERG11* observed not only in azole-resistant strains, but also in azole-susceptible ones. Therefore, it is possible that other molecular mechanisms might be involved in the development of the resistant phenotype. These mechanisms could be efflux pumps, other mutations, or overexpression of genes involved in the biosynthesis of ergosterol (Lamping et al. 2009).

In addition to the mutations in *ERG11* gene, also overexpression has been reported to be involved in the resistance phenotype of *Candida*species. Multidrug resistance (MDR) proteins are of efflux pump transporters, including the adenosine triphosphate-binding cassette (ABC) transporters and the major facilitator super family. Also, in *Candida* species the efflux pump genes associated with azole resistance include *Candida* drug resistance (*CDR*)*1* and *CDR2*, and MDR (Coste et al. 2004, Morio et al. 2010).

He et al. (2015) studied the molecular mechanisms responsible for itraconazole resistance in clinical isolates of*C. krusei* and found *ERG11* gene polymorphisms that may not be involved in the development of itraconazole resistance in *C. krusei*, but overexpression of *ERG11* and*ABC2* might be responsible for the acquired itraconazole resistance of the clinical isolates.

Future studies using cloning and induction of such nonsynonymous mutations might clarify the mechanism of reduced azole sensitivity observed in this study.

This study revealed novel synonymous and nonsynonymous mutations in*Candida* species known to be resistant to fluconazole, itraconazole, and voriconazole. The results suggest that the Y166S mutation found in an isolate of *C. krusei* with dose dependent sensitivity to voriconazole may be responsible for its reduced susceptibility to azoles. The mutation might act by affecting the functionality of 14α-demethylase. Due to limited number of experimental strains, further studies are needed to confirm this hypothesis, for example, novel experiments with other strains with reduced susceptibility to voriconazole such as induction of mutation and evaluation of gene expression related to resistance.

If confirmed these results, they could contribute in the developing strategies to understand and solve the problem concerning to the resistance, and one of the alternatives are the new prospections of bioactive molecules with antifungal activity based on the genetic and molecular characterisation of the isolates, making possible to offer besides the socioeconomic, technological, and industrial viability, an appropriated treatment based on the best specificity of new molecule activity, mainly in emergency cases of resistant isolates.
